# Movement within foot and ankle joint in children with spastic cerebral palsy: a 3-dimensional ultrasound analysis of medial gastrocnemius length with correction for effects of foot deformation

**DOI:** 10.1186/1471-2474-14-365

**Published:** 2013-12-23

**Authors:** Peter A Huijing, Menno R Bénard, Jaap Harlaar, Richard T Jaspers, Jules G Becher

**Affiliations:** 1 Move Research Institute Amsterdam, and Faculteit der Bewegingswetenschappen, Vrije Universiteit Amsterdam, Van de Boechorststraat 9, 1081 BT, Amsterdam, The Netherlands; 2 Move Research Institute Amsterdam, and Department of Rehabilitation Medicine, VU Medical Center, Amsterdam, The Netherlands

**Keywords:** Cerebral palsy, Ultrasound imaging, m. gastrocnemius medialis, Ankle joint, Ankle moment, Foot deformation

## Abstract

**Background:**

In spastic cerebral palsy (SCP), a limited range of motion of the foot (ROM), limits gait and other activities. Assessment of this limitation of ROM and knowledge of active mechanisms is of crucial importance for clinical treatment.

**Methods:**

For a comparison between spastic cerebral palsy (SCP) children and typically developing children (TD), medial gastrocnemius muscle-tendon complex length was assessed using 3-D ultrasound imaging techniques, while exerting externally standardized moments via a hand-held dynamometer. Exemplary X-ray imaging of ankle and foot was used to confirm possible TD-SCP differences in foot deformation.

**Results:**

SCP and TD did not differ in normalized level of excitation (EMG) of muscles studied. For given moments exerted in SCP, foot plate angles were all more towards plantar flexion than in TD. However, foot plate angle proved to be an invalid estimator of talocrural joint angle, since at equal foot plate angles, GM muscle-tendon complex was shorter in SCP (corresponding to an equivalent of 1 cm). A substantial difference remained even after normalizing for individual differences in tibia length. X-ray imaging of ankle and foot of one SCP child and two typically developed adults, confirmed that in SCP that of total footplate angle changes (0-4 Nm: 15°), the contribution of foot deformation to changes in foot plate angle (8) were as big as the contribution of dorsal flexion at the talocrural joint (7°). In typically developed individuals there were relatively smaller contributions (10 -11%) by foot deformation to changes in foot plate angle, indicating that the contribution of talocrural angle changes was most important.

Using a new estimate for position at the talocrural joint (the difference between GM muscle–tendon complex length and tibia length, GM relative length) removed this effect, thus allowing more fair comparison of SCP and TD data. On the basis of analysis of foot plate angle and GM relative length as a function of externally applied moments, it is concluded that foot plate angle measurements underestimate angular changes at the talocrural joint when moving in dorsal flexion direction and overestimate them when moving in plantar flexion direction, with concomitant effects on triceps surae lengths.

**Conclusions:**

In SCP children diagnosed with decreased dorsal ROM of the ankle joint, the commonly used measure (i.e. range of foot plate angle), is not a good estimate of rotation at the talocrural joint. since a sizable part of the movement of the foot (or foot plate) derives from internal deformation of the foot.

## Background

Children with cerebral palsy (CP) show disorders of movement and posture as a consequence of non-progressive disturbances in the infant brain [1]. This results in limitations in activities and mobility problems preventing optimal participation in daily life. With a prevalence of about 2 per 1000 live births in Western countries, CP is the most common cause of physical disabilities in pediatric rehabilitation medicine [2]. Spastic CP (SCP) is the most common motor disorder and compromises gait of about 80% of these children [3].

A limited range of motion of the foot (ROM), resulting in abnormal gait and limitation of activities, is a common problem for such children [4,5]. A better understanding of mechanisms active in reduced ankle ROM in SCP is essential to allow possible optimization of treatment. A major problem in attaining better understanding of mechanical problems at the ankle joint is unavailability of clear reference conditions in terms of either muscle-tendon complex length or muscle force.

Often, research on the human spastic calf has been primarily focused on GM muscle belly (for review, see [6]). To our knowledge, there are no studies relating joint angle and muscle-tendon complex length in SCP.

A major improvement in experimental approach has been to combine ultrasound measurements of gastrocnemius muscle with measurement of external moments exerted at the ankle [7-9], as this provides crucial constraints on mechanical conditions around the ankle, allowing more fair comparisons of SCP and TD children. Combining these techniques some authors attributed mechanical effects at the SCP ankle to shorter muscles [10] or to muscles with shorter fascicles [8,11,12].

Note that in all these cases, and also in other clinical and fundamental human movement research, ankle joint angle is estimated as the angle between elements of the lower leg (e.g. tibia or fibula) and the foot sole (or ground, or foot–plate respectively). Clinically it is known that particularly in SCP deformation of the foot is obtained easily, but to our knowledge, effects of that have not been studied systematically

Therefore, our present aim was to study ankle ROM as function of externally applied moments and measure GM muscle-tendon complex lengths in SCP and TD children. Data thus obtained will allow us to test the following hypothesis: At equal foot plate angles, GM muscle-tendon complex lengths (absolute or normalized for tibia length) are equal for SCP and TD children.

## Methods

### Subjects

The ages of participating children ranged from nine to thirteen years. Eight children with spastic cerebral palsy (SCP) and sixteen typically developing (TD) children participated in this study with informed consent from their parents (as well as from the children themselves, if aged 12 years or over). The study was approved by the Medical Ethics Committee of the Vrije Universiteit Medisch Centrum.

For a summary of individual clinical characteristics of SCP subjects see Table 1.

**Table 1:** Individual clinical characteristics of SCP subjects

**Patient #**	**Sex**	**Age**	**Hemi-or di- plegia**	**GMFCS (according to Palisano, ref **[13]**)**	**Clinical characteristics of foot of the loaded tested leg (standing)**
05	M	10.9	2	1	normal
06	M	11.5	2	3	Valgus: light
07	M	11.0	2	3	Valgus: serious
08	F	9.2	2	2	Valgus: serious
09	M	10.1	2	4	Valgus: serious
10	F	12.0	1	1	Valgus: serious
17*	M	12.9	2	3	Valgus: serious
19	M	10.9	2	2	Valgus: serious

*X-rayed subject. All feet had a full range of motion from varus to valgus of the calcaneus and suppination to pronation of the forefoot (i.e. no fixed deformation).

All SCP children were indicated clinically for treatment of reduced ankle ROM, due to a fixed plantar flexor contracture. This was defined clinically as a foot sole dorsal flexion lower than 90° with the tibia, with full knee extension. SCP children had been subjected neither to prior surgical interventions at the lower limbs, nor to Botulineum toxin-A treatment within up to half a year prior to measurement.

### Anthropometry

For both legs of SCP children, ankle range of motion (ROM) towards dorsal flexion was measured manually and the leg with the lowest ROM was selected for further study.

For TD children, the right leg was studied.

The children were lying prone on a bench, with both feet (without apparatus) overhanging the edge of the table. In agreement with clinical practice, this position is used as a reference one. If this reference foot-tibia angle could not be attained, the nearest similar position was taken for this particular measurement. Tibia length and distance between the malleoli and the Achilles tendon were measured. Tibia length (*ℓ*_*(tib)*_) was calculated as the mean distance from the tibia plateau to the most prominent part of the malleolus, measured at both the medial and lateral side of the leg. Malleolus height was calculated as the mean perpendicular distance from the most prominent part of the lateral and medial malleolus to the foot sole.

### Moment - foot-plate angle data

To manipulate the angle between the foot sole and the tibia (foot-plate angle), a custom designed hand-held dynamometer was used, that allows individual correction for non-rigid varus/valgus foot forefoot (for details see [14]). The purpose of applying such individual changes to the foot plate was to limit as much as possible variation between individual subjects in movement executed, due to variation in participation of the subtalar joints in the dorsal and plantar flexion movements imposed via the hand held dynamometer.

To obtain such limitation, the subtalar joints need to be stabilized as much as possible: This was obtained by applying the following corrections:

(1) The calcaneus is rotated to the neutral position within the frontal plane. This in fact brings the calcaneus to a neutral position under the tibia.

(2) Adduction of the forefoot is applied with the aim of bringing the calcaneus midline to pointing between the 2nd and 3rd ray of the forefoot. This will cause the caput of the talus to protrude under the skin. If the talocalcaneal joint is sufficiently stable the skin over the most medial part of the talus will wrinkle on imposing dorsal flexion.

(3) If the talocalcaneal joint is not sufficiently stable as yet, the fore- and midfoot are supinated until sufficient stability is reached.

**Figure 1:**
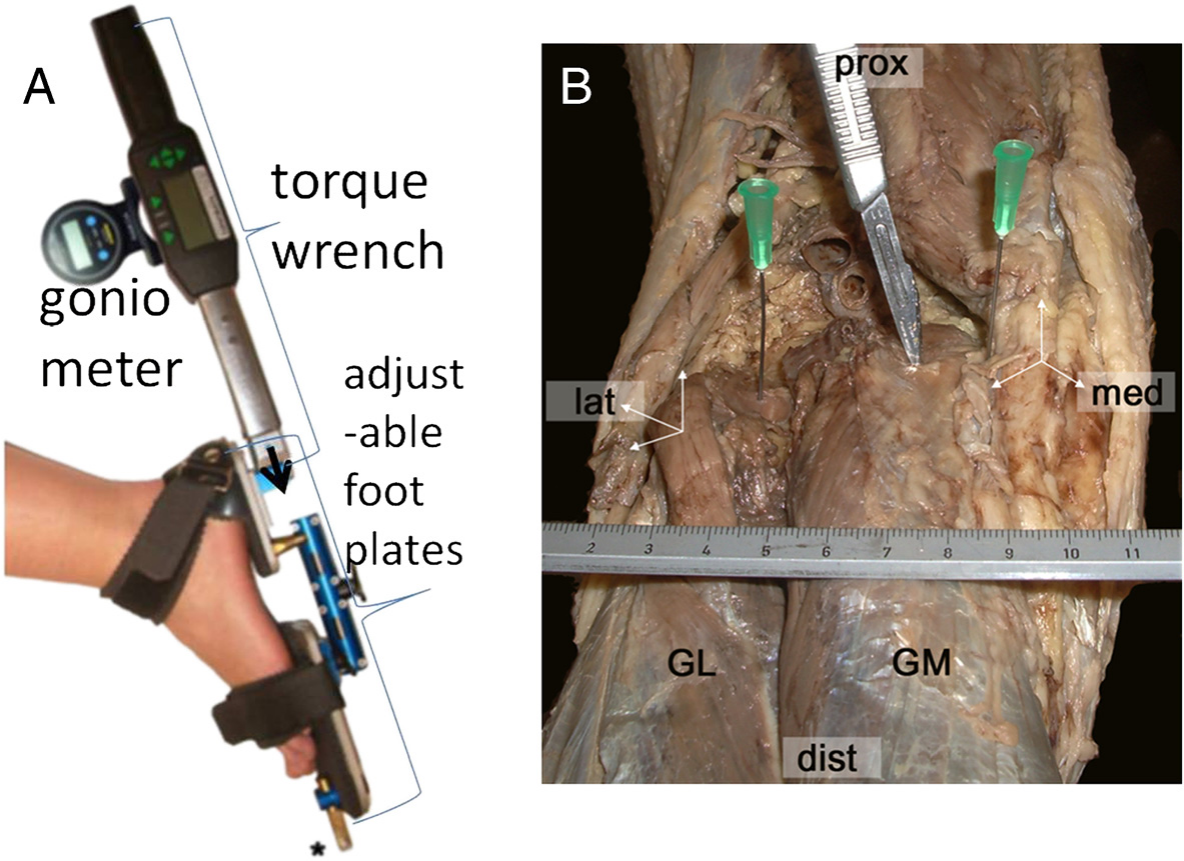
**Moment and angle measurement set up and example of dissected origin of human gastrocnemius muscle and glenoid cavity. A**. The foot strapped to the foot plates, to which also the torque wrench is attached. The gonio meter is used for measuring the angle with the horizontal. The tip of the arrow indicates the location of a reference point used for quantifying the moment measured. **B**. Medio-dorsal view of the dissected of human cadaver popliteal fossa and gastrocnemius muscle. Two needles (green attachments) are inserted on the most prominent point in dorsal direction of lateral and medial condyles, respectively. The tip of the scalpel inserted indicates the target point for describing the path of GM. Note that by taking this point as the closest obtainable estimate for the origin of GM a small length of GM proximal to the marked point is neglected in SCP and TD subjects. The ruler indicates a scale of cm and mm.

The dynamometer for measurements of externally applied moments [Nm] is equipped with an inclinometer (goniometer that measures angle with the horizontal) (Figure 1A). If care is taken to position the tibia horizontally by supporting the lower leg on the table, the inclinometer indicates the angle between foot plate and tibia (*φ*_*fp*_) within the sagittal plane (i.e. angles of dorsal and plantar flexion, with positive values referring to dorsal flexion conditions).

Foot plate angle data were collected during exertion of a moment of: a) -4 Nm, b) 0 Nm, and c) +4 Nm, respectively, as well as moments data collection at angles intermediate between a) and b); and c) and d), respectively.

All such dynamometer measurements were repeated five times and for each repetition the plateau values of moments applied were held for five seconds [14]. The mean of five repetition values at the end of the five seconds holding time was taken as a data point for a subject.

### 3-D ultrasound imaging

Ultrasound scans of GM were made using a B-mode ultrasound device (Technos MPX, ESAOTE S.p.A., Italy), with a 5 cm linear array probe (12.5 MHz). The ultrasound images were sampled at 25 Hz using a video card (miroVIDEO DC30; Pinnacle Systems Inc.).

Prior to executing the protocol, the setup was calibrated by tracking, within the ultrasound image, a cross-point of two wires located in a water cube whilst moving the ultrasound probe around the cross-point [15]. This way, the system was calibrated spatially for three translations and three rotations. Using this calibration, 3D coordinates of pixels within ultrasound images were calculated using software custom programmed in Matlab (version 7.1, the Mathworks Inc., 2005).

The scans were made as follows: (1) An area for a scanning path was outlined on the skin (markers indicating the palpated edges of lateral and medial condyles of the femur, the lateral and medial borders of GM muscle belly, the most distal end of GM muscle belly and the calcaneus, (2) the skin surface over the tissue volume to be scanned was covered by a thick layer of ultrasound gel (approximately 5 mm) to improve image quality; (Figure 1B); (3) the ultrasound probe was moved with an orientation to image cross-sections of GM; (4) on average a scan took approximately thirty seconds; (5) the position and orientation of the ultrasound probe were sampled at a frequency of 25 Hz by tracking a three marker frame, rigidly attached to the probe, using one camera Optotrak 3020 system (Northern Digital, Waterloo, Canada).

On the basis of each 3-D voxel array, the scanned volume of GM was reconstructed. This array was used in further analysis to calculate a reconstructed image of the mid-longitudinal plane of GM. This method has the advantage that the proper plane containing real fascicles can be selected post-experimentally. Proper selection of this plane is crucial for valid measurements (for details see [16]). Such a reconstruction of GM muscle was made for each of the five moment-angle conditions.

To estimate the most proximal point of GM to be used in the analysis of the reconstructed ultrasound image is based on an anatomical validation (see below).

The most distal point for the image analysis of GM belly was obtained by marking the distal end of the most distal fiber and the middle of the attachment of the Achilles tendon on the calcaneus. For details on such measurements within the reconstructed midlongitudinal image (see [17]).

### Anatomical validation of measurement points within ultrasound images

The most distal end of GM belly and the point of most proximal attachment of the Achilles tendon on the calcaneal bone on are well discernible within the ultrasound image. Ideally, the true origin of GM would also be selected within such image. However, GM as it approaches its origin curves across the medial condyle to go deep into the popliteal fossa to reach its true origin. It should be noted that in ultrasound imaging this point of origin is not discernible. Therefore, a new proximal reference point along the path of GM needs to be defined that will serve in defining the target plane, as well as play a role in estimation of muscle-tendon complex length (*ℓ*_*m+t*_). Figure 1B shows an example of dissections performed and measurements of such a target point relative to structures discernible also in ultrasound images (i.e. edges of medial and lateral condyles). For seven human cadavers, this distance), normalized for intercondyle distance, between this point (Figure 1B) and the lateral condyle, was found to be 0.74 ±0.08 (mean ± S.D).

In compliance with Dutch law, the Faculteit der Geneeskunde, in collaboration with the Faculteit der Bewegingswetenschappen, of the Vrije Universiteit Amsterdam, have a dissection room for educational and research purposes. To obtain human cadavers, for such use, a program is run that accommodates the wish of individuals to arrange for the donation of their body for scientific purposes after their death (willed body program for adult Dutch citizens). The human cadavers used in the present study were obtained from this source.

### Electromyography

To quantify excitation during the dynamometer and ultrasound protocol, surface electromyographic (EMG) signals of tibialis anterior muscle (TA) and gastrocnemius lateralis muscle (GL) were digitized at 1000 Hz using a multichannel system (Porti5, TMS-International™, The Netherlands). Skin preparation and EMG electrode placement were carried out according to SENIAM guidelines [18]. EMG at maximal voluntary contraction (MVC) (duration about 5 seconds) was measured in prone position for both muscles at a foot plate angle of 0° (or maximal dorsal flexion in SCP if 0° could not be attained). Off-line, EMG signals were high-pass filtered at 20Hz to remove artifacts, and normalized with respect to the peak value of the EMG during MVC. If EMG signals exceeded 10% of MVC, moment-angle measurements, as well as ultrasound scans were excluded from the analysis [19].

### Data analysis

Length variables of GM muscle were measured using an image analyzing tool, custom programmed in MATLAB (version 7.1, the Mathworks Inc., 2005). Using this analyzing tool, the mid-longitudinal plane of GM was defined within the 3-D voxel array. Within this selected plane, muscle variables were measured by a single experimenter: 1) Muscle-tendon complex length was calculated as the sum of the length of GM belly and length of external tendon. These measurements were repeated five times on each reconstructed image and mean results used as a data point for the individual. For further details, concerning these measurements and calculations see [16,17].

The complete interactive analysis was repeated five times on each reconstructed image, and means were calculated for all muscle variables. A pilot study showed that with more than five repetitive measurements, standard deviations of the variables for each individual did not decrease further and were less than 5% for the length variables.

### Joint configuration data of ankle and foot in X-ray images

To view joint configurations of ankle and foot in the sagittal plane, X-ray images were taken of the foot and distal part of the lower leg. These images were made with subjects lying on their right side. The X-ray source was located medially to the ankle and the photographic plate laterally (i.e. mediolateral projection). The foot was placed in the foot-fixation and images were made at foot plate angles corresponding to those measured at 0 Nm and +4 Nm.

Permission was obtained to X-ray one child of the SCP group (age = 13 y, body mass = 60 kg, *ℓ*_*(tib)*_ = 38 cm). As taking X-rays on TD children was judged not to be ethical, they were taken on two typically developed adult volunteers (members of the research group, not participating in the main part of the study).

X-ray images were analyzed twice by superimposing the 0 Nm and +4 Nm images for each subject by adjusting to identical magnification and aligning: (1) the fibula and (2) the talus. The former pair shows total movement of the footplate with concomitant movements of the muscle insertions within the foot and the latter shows movement of the tibia ascribable to rotation within the talocrural joint as well as movement of the foot and its constituting parts due to deformation of the foot.

In both types of aligned images, muscular length changes were estimated by drawing a rectangle perpendicular to the estimated line of pull of a muscle at the insertion (at 0 Nm) and determining its relevant size needed to just reach the new location of the same insertion (at +4 Nm). The bony parts used for these estimates were: the most dorsal and proximal edge of the calcaneus, the most plantar and dorsal edge of tuberositas of metatarsalis V and the most plantar and dorsal edge of the basis of metatarsale I, as insertions for GM, m. peroneus brevis (PB) and m. tibialis anterior (TA), respectively.

### Statistics

A Student’s t-test was used to test for significant differences between SCP and TD regarding: age, body mass, tibia length, and malleolus height.

A two way ANOVA with one between subjects factor (experimental group) and one within subjects factor (repeated measurement: externally applied moment or footplate angle (*φ*_*fp*_) or GM relative length) was used to test for main effects of and their possible interaction on GM *ℓ*_*m+t*_, and on GM *ℓ*_*m+t*_, normalized for *ℓ*_*(tib)*_ or *φ*_*fp*_. In case of interaction effects, bonferroni post hoc tests were performed to further locate differences. Two way ANOVA was used to test for differences of EMG (normalized for MVC values) of TA and GL measured during dynamometry measurements and during collection of ultrasound scans of GM to test for effects of experimental *group* (SCP–TD differences*)* and externally applied *moment* (repeated measurement). For all statistics, the level of significance was set at p < 0.05.

## Results

### Subject variables: SCP and TD

Eight SCP children participated in this study. Criteria for inclusion were plantar flexion contracture and no treatment for at least 180 days. Note that 2 SCP children were included without having previous treatment. Several SCP children (n = 6) received treatment (botulinum toxin, n = 5), mean 1146 days before this experiment), or botulinum toxin plus rhizotomy (n = 1, 180 days before the present experiment, Table 2). Note, however, that all subjects still met inclusion criteria.

**Table 2:** Descriptive and anthropometric variables of subjects

	**TD (n = 16)**			**SCP (n = 8)**	
**Variable**	**Mean**	**SE**	**Sign.**	**Mean**	**SE**
Age [years]	11.3	0.2	^**#**^	11.1	0.3
Body mass [kg]	39.1	1.6	^**#**^	40.8	3.5
Tibia length [cm]	35.3	0.8	^**#**^	32.6	1.0
Malleolus height [cm]	6.7	0.1	^*^	5.8	0.2
Time since prior treatment (n = 6, 2 no treatment) [days]	-	-	-	918	Range 180–1682

* = sign difference (p < 0.01), ^#^ = not sign (p > 0.05), AT: Achilles tendon.

Mean body parameters are shown in Table 2. Note that mean age, mean body mass, and Achilles tendon moment arm of SCP and TD were not different. Although mean tibia length was 2.7 cm lower in SCP, this could not be shown to be significantly different from TD at group level (p = 0.08).

Note that only malleolus height (measured without foot plate and no external moments exerted by the researcher) differed significantly (p < 0.01) between the groups. Malleolus height with respect to the foot plate was approximately 0.9 cm lower in SCP (Table 2), suggesting differences in configuration of the bones constituting the foot. We conclude that analyzing such differences between TD and SCP groups is crucial for an adequate interpretation of muscle geometrical data, as well as foot-plate angle-moments data, because they may not only affect ankle and foot function, but also muscle-tendon complex length.

### Degree of excitation of muscles

A potential explanation for differences in moment-foot-plate angle characteristics are differences in levels of activity in plantar and dorsal flexor muscles, related to the impairment. However, for both GL and TA, no main effects were shown (ANOVA: TD vs SCP group and moment) on normalized EMG (mean ± SE values for TA and GL were 3.15 ± 0.33% and 3.29 ± 0.32% for TD and 3.20 ± 0.84% and 2.86 ± 0.65% for SCP, respectively). It is concluded that any differences in moment-foot-plate angle or length-moment characteristics should be ascribed to factors other than degree of excitation of muscles.

### Muscle-tendon complex length as a function of foot plate angle

If SCP and TD children would have identical skeletal dimensions, absolute *ℓ*_*m+t*_ would be expected to be identical at equal foot plate angles. In contrast to such expectation, for the range of overlap of foot plate angles between the two groups (i.e. from -51° to -10°), *ℓ*_*m+t*_ was significantly lower in SCP than in TD children. For example, at *φ*_*fp*_ = 28°, *ℓ*_*m+t*_ was 3.6 cm smaller in the SCP group. Note that to attain this foot plate angle in SCP, external moment applied was approximately 2 Nm higher than in TD.

**Figure 2:**
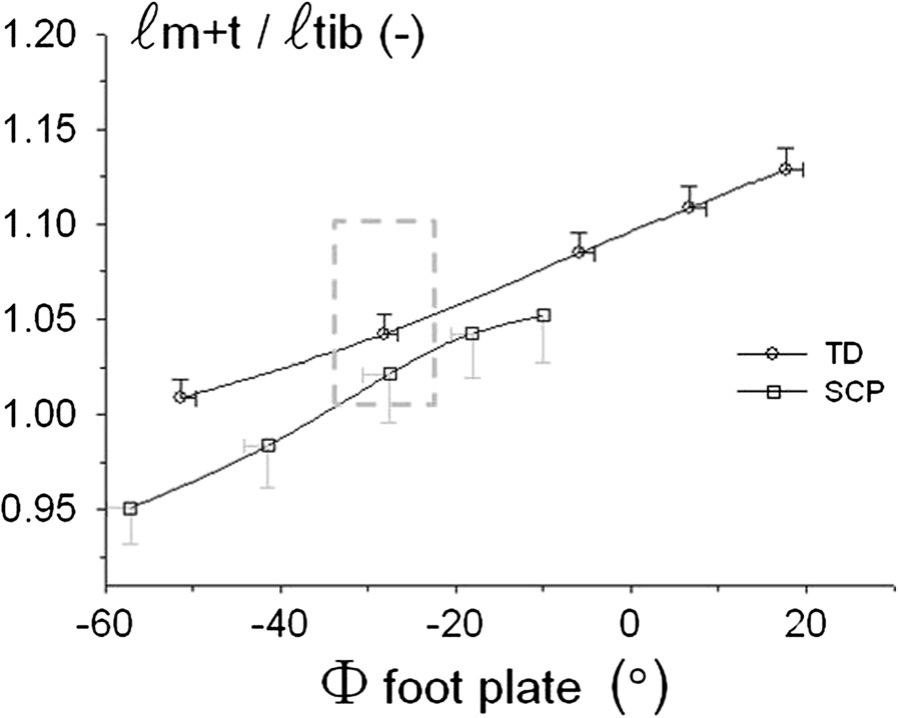
**SCP-TD comparison: GM muscle tendon complex length.** Length of the muscle-tendon complex (*ℓ*_*m+t*_), normalized for tibia length, plotted as a function of foot plate angle. Mean values (and SE) and third-order polynomial fitted curves are shown. (φ_foot plate_) is represented as deviation from the neutral position (0°), i.e. with the longitudinal axis of the tibia perpendicular to the foot plate. Note that normalized length is even different for a point at similar foot plate angle (indicated by a dashed rectangle).

Figure 2 shows that values for GM muscle-tendon complex length (*ℓ*_*m+t*_), even if normalized for tibia length, in SCP and TD groups are not similar at similar foot plate angles. This indicates very important, functional and morphological differences, between the groups. For a common foot plate angle data point, GM muscle-tendon complex length after normalization (i.e. *ℓ*_*m+t*_/*ℓ*_*(tib)*_), was 1.02 versus 1.04 times tibia length for SCP and TD children, respectively (Figure 2). This corresponds to a remaining substantial absolute difference in mean GM length (difference *ℓ*_*m+t*_ = 0.8 cm, with bigger differences suggested at other foot plate angles). It is concluded that increasing the dorsal flexion moment at the ankle in SCP does not induce as high angular changes at the talocrural joint as in TD. Such differences should be explained. In agreement with differences in malleolus height, and characteristics of the deformed feet of SCP children (Tables 1 and 2), a likely explanation is the presence of differences between the SCP and TD foot. Particularly, differences in deformation of the foot upon applying moments seem likely. In addition, a search is indicated for a better estimator of events at the ankle joint and their effect on triceps surae length to allow fair comparison of TD-SCP muscle.

### Analysis of some X-ray images of ankle and foot

The arguments presented above raise the question: If triceps surae length changes are much more limited in SCP than in TD children, how is the increased foot plate angle (Figure 2) attained. Above we hypothesized that deformation of the foot is a likely mechanism. To asses if we could find any evidence in support of that concept, we needed images that would allow us to view such events at ankle joints and other joints of the foot. For one SCP child and two typically developed adults, we were allowed access to X-ray images, taken at two foot plate angles corresponding to those attained on exertion of 0 Nm and +4 Nm.

### SCP

**Figure 3:**
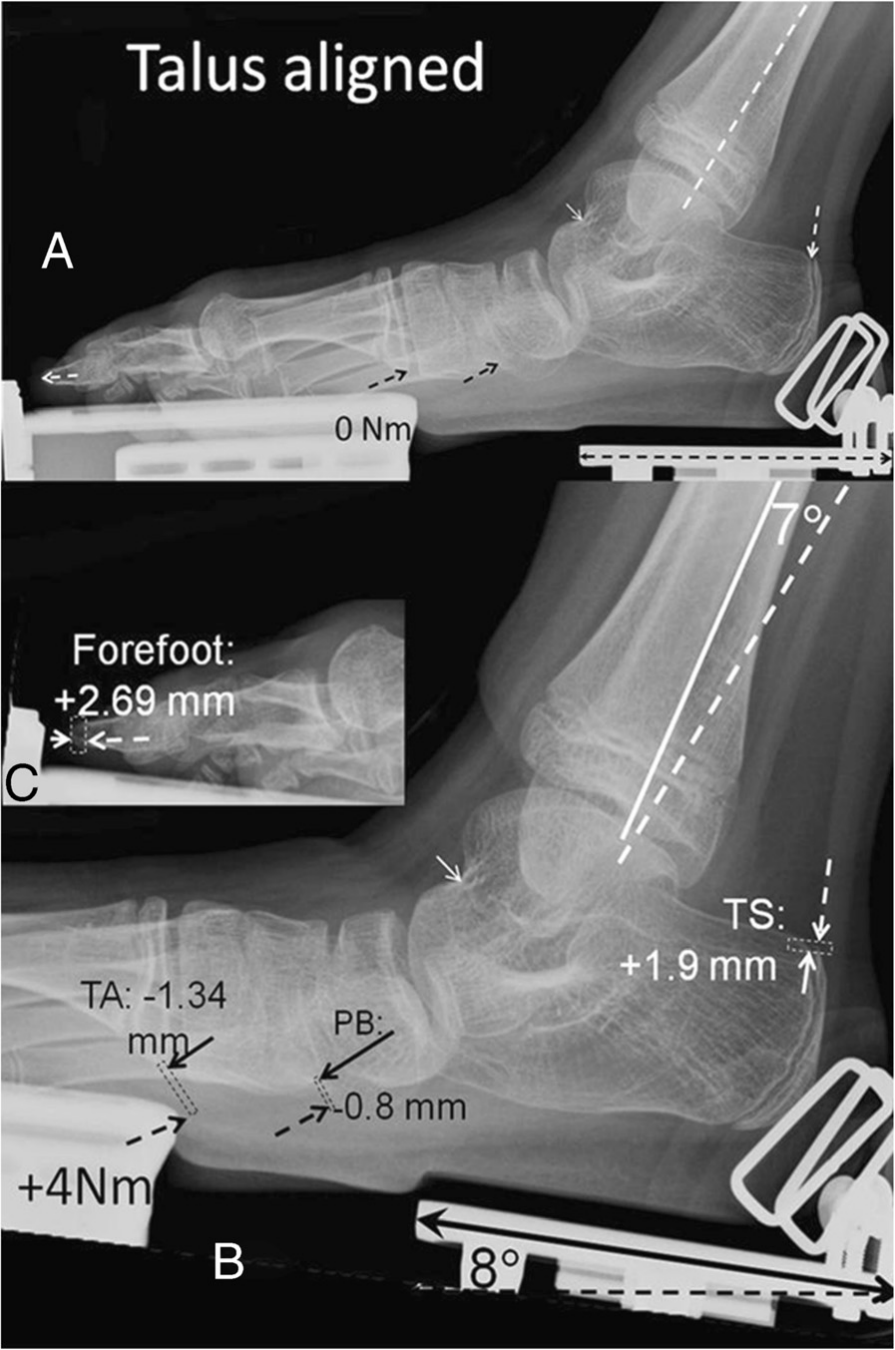
**Analysis of X-rays for a SCP subject after talus alignment. A**. The foot in a position similar to that when exerting 0 Nm. Under high magnification markers (dashed lines and arrows) were placed for alignment and at particular insertion points of muscles and along the fibula. Note that, for example, at the navicular bone, subluxation and an extreme position (gapping) of the calcaneo-navicular-joint is noticeable. **B**. The foot in a position similar to that when exerting 4 Nm dorsal flexion. The talar bone was aligned with that in **A**. Such talar bone alignment allows distinction of contributions to foot plate movement and muscular length changes by foot deformation and crural bone movements, respectively. The markers of **A**. were copied to image **B**. (dashed lines or arrows). New markers (solid lines or arrows) placed on the reference points in the new condition. Length changes were estimated (rectangles) for GM (m. triceps surae), TA (m. tibialis anterior) and PB (m. peroneus brevis) and angular changes for the fibula and foot plate. Note that, for clarity, magnification of A. and B. are not identical. The arrow on the heel part of the foot plate is used for calibration (actual dimension = 8.98 cm). **C**. Inset of the forefoot to show the presence foot lengthening upon exertion of +4 Nm.

Despite the absence of sideways support of the foot when exposing the X-ray, the tibia and fibula are well aligned in mediolateral direction with each other and with the talus, so that the fibula is seen near the longitudinal center of the tibia and the malleoli overlap in the X-ray. Radiological experience indicates that, if the foot is not supported laterally, an exorotation of crural bones with respect to the foot is expected (see also Figure 3). Therefore, for this SCP child, this seems to indicate the presence of torsion in the tibia, with its distal end rotated medially also its planovalgus foot is apparent.

The talus, instead of its normal cranial position with respect to the calcaneus, is positioned rather medially to it (subluxation). Note that the alignment of articular surface of the talus with tibia seems normal (Figure 3). On exerting dorsal flexion moments on the footplate, the relative position of talus and calcaneal bones changed by small amounts only. Therefore, we conclude that our foot-fixation mechanism is effective in limiting (but not always fully preventing) movement within the subtalar joints, and thereby limiting potentially confounding individual variation between SCP individuals for this experiment.

Values for total change of the foot plate angle, as well as concomitant muscular length changes were obtained from fibula aligned images (Table 3). Superimposing the talus within the two X-ray images of this SCP patient allows distinction of contributions to foot plate rotation of movement of the talocrural joint from those of subtalar joints of ankle and other joints of foot. In such analysis, dorsal flexion at the talocrural joint constituted only 47% of foot plate dorsal flexion rotation, the remainder ascribable to deformation of the foot. Therefore, for this SCP child it is confirmed that foot deformation and foot pad compression allow for substantial rotation of the foot plate and that if the joints of the foot would be very stiff, the movements of the crural bones with respect to the foot plate would be more than halved.

**Table 3:** Summary of results obtained from X-ray images

**Subject**	**Rotations 0 to +4 Nm**		**Muscle-tendon complex length changes 0 to +4 Nm**					
	**Foot plate angle with tib-fib (º)**	**True ankle dorsal flexion (º)**	**∆*ℓ ***_***triceps surae***_		**∆*ℓ ***_***peroneus brevis***_		**∆*ℓ ***_***tibialis anterior***_	
			Total* (mm)	***Due to foot deform.‡ (mm)***	Total* (mm)	***Due to foot deform.‡ (mm)***	Total* (mm)	***Due to foot deform.‡ (mm)***
SCP	15.0	7.0	+ 8.8	+1.9	-1.3	-0.8	-6.7	-1.3
TD A.	15.0	13.5	+12.8	≈0.0	+6.0	+3.0	-8.6	≈0.0
TD B.	18.0	16.0	+13.2	≈0.0	+6.3	+2.5	-6.0	≈0.0

*obtained from fibula aligned images; ‡ obtained from talus aligned images.

### TD

Because of the absence of sideways support of the foot during exposure of the X-ray, for the TD subjects, the tibia and fibula are exorotated with respect to the foot. This a typical result for the conditions imposed. Note that this is unlike results for the SCP child studied indicating differences in torsion grown into the crural bones.

**Figure 4:**
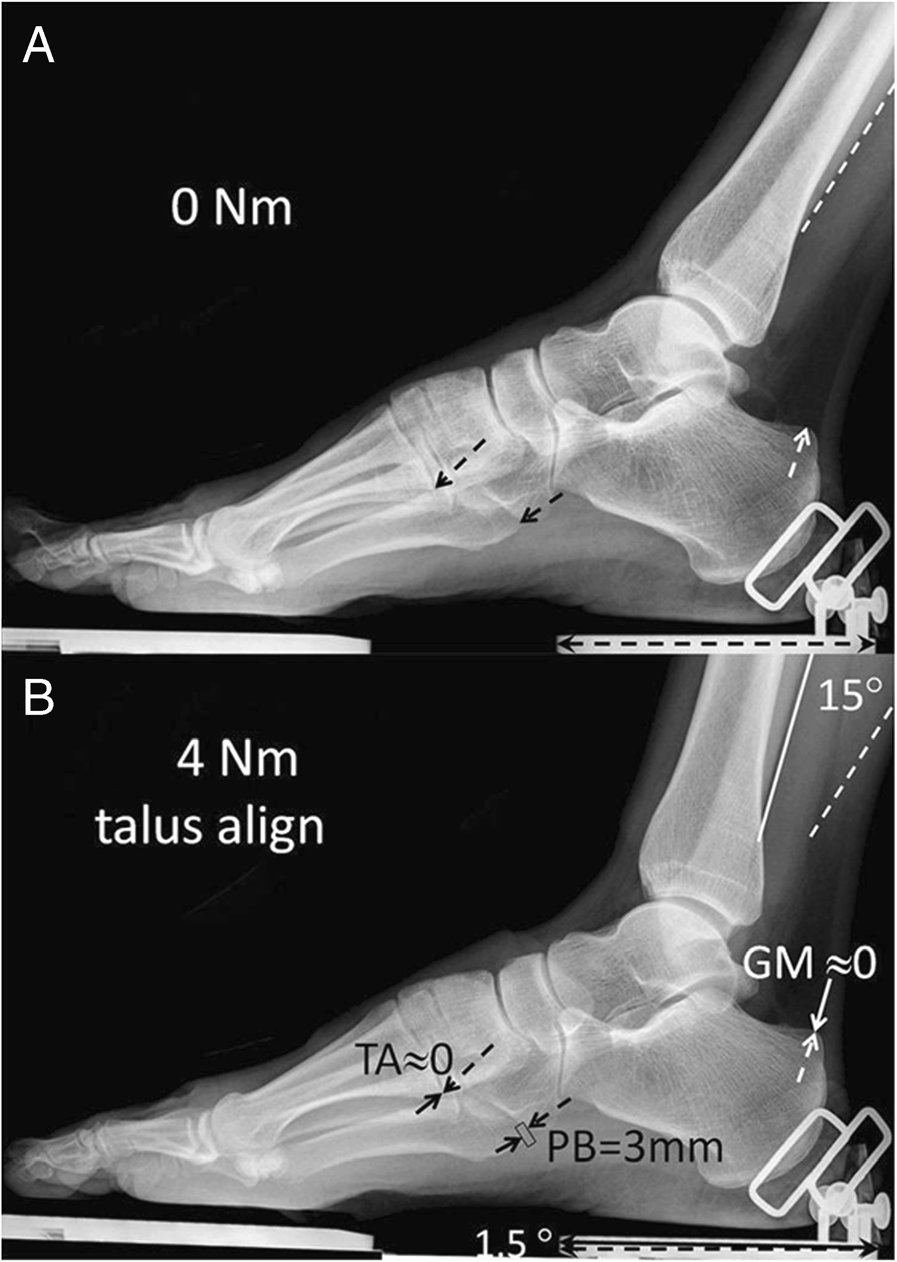
**Analysis of X-rays for a TD adult subject after talus alignment. A**. The foot in a position similar to that when exerting 0 Nm. Under high magnification markers (dashed lines and arrows) were placed for alignment and at particular insertion points of muscles and along the fibula. **B**. The foot in a position similar to that when exerting 4 Nm dorsal flexion. The talar bone was aligned with that in A. Such talar bone alignment allows distinction of contributions to foot plate movement and muscular length changes by foot deformation and crural bone movements respectively. The markers of **A**. were copied to image **B**. (dashed lines or arrows). New markers (solid lines or arrows) placed on the reference points in the new condition. Length changes were estimated (rectangles) for GM (m. triceps surae). TA (m. tibialis anterior) and PB (m. peroneus brevis) and angular changes for the fibula and foot plate, as well as calcaneal angle (not shown). The arrow on the heel part of the foot plate is used for calibration (actual dimension = 8.98 cm).

For the typically developed adults, at a foot plate angle corresponding to exerting 0 Nm, the acute angle between calcaneus and foot plate was positive (e.g. Figure 4A) reflecting the presence of a longitudinal arch of the foot. Applying +4 Nm on the foot-plate has the following effects: The foot plate rotates towards dorsal flexion, of which a sizable fraction (i.e. 89-90%) is caused by true ankle dorsal flexion (Figure 4B, Table 3). It is concluded that also in TD adults foot plate angle is likely not to be a valid indicator of angular changes at the talocrural joint. However, errors made are much smaller than in the SCP child, but remain substantial, as the foot plate rotation due to tarsus deformation still constitutes between 10-11% of dorsal flexion at the talocrural joint.

### In search of a better estimate of talocrural joint angle

**Figure 5:**
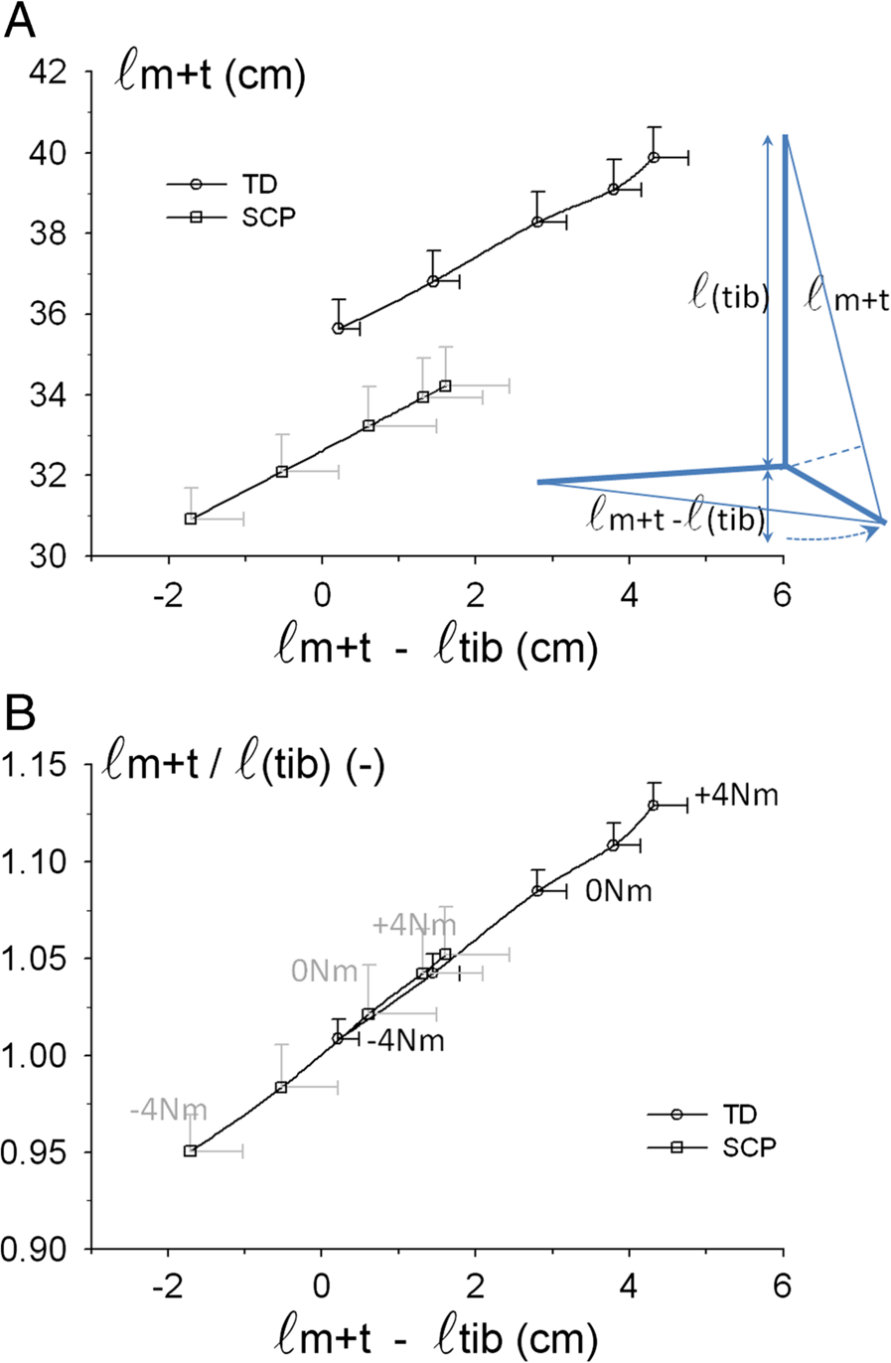
**SCP-TD comparisons for absolute and normalized GM length.** Inset: Schematic illustrating the concept of GM relative length with respect to tibia length (*ℓ*_*m+t -*_*ℓ*_*(tib)*_) as a (co-)determinant of ankle joint angle. **A**. Absolute GM muscle-tendon complex length (*ℓ*_*m+t*_). **B**. GM muscle-tendon complex length normalized for tibia length (*ℓ*_*m+t*_/*ℓ*_*(tib)*_) GM relative length (*ℓ*_*m+t -*_*ℓ*_*(tib)*_), as estimate of talocrural joint angle changes, is plotted as independent variable. For a number of data points the external moment applied to attain such positions is indicated (black font for TD and gray font for SCP, respectively). Additional statistical analysis was performed on fitted data for the overlapping GM relative lengths (i.e. equal lengths: +0.33 > *ℓ*(m + t)-*ℓ*(tib) < +1.61). Note that very different moments were applied externally for that range. Also note that GM in SCP generally operates at lower relative lengths. Mean values + SEM are plotted.

We tested the variable (*ℓ*_*m+t -*_*ℓ*_*(tib)*_) as a better estimator of events at the ankle joint (for a schematic illustrating this idea, see inset Figure 5A). Below, we will refer to this variable as “GM relative length”.

Figure 5 shows that plotting absolute GM muscle-tendon complex length (*ℓ*_m+t_) as a function of GM relative length still shows expected substantial differences between SCP and TD groups. Note that the similarity of slopes (Figure 5A) in the overlap area between is an indication for similarity of moment arms for the TD and SCP groups.

Normalization of individual *ℓ*_*m+t*_ values for individual tibia length removes completely the differences in muscle-tendon complex length for the overlapping ranges of relative GM length, so that this variable of SCP and TD groups is described by one function. However, a number of differences remain noteworthy (Figure 5): (1) Despite equal GM relative lengths within the overlapping length range, corresponding moments exerted at the ankle differ substantially (marked for reference in Figure 5B). (2) Overall, SCP triceps surae operates at significantly lower GM relative lengths than TD (mean values). (3) Also note that the range of GM relative lengths over the full range of externally exerted moments (i.e. (*ℓ*_*m+t(+4Nm)-*_*ℓ*_*(tib)*_)-(*ℓ*_*m+t(-4Nm)-*_*ℓ*_*(tib)*_)) is significantly smaller in SCP than in TD (i.e. mean ± SE 3.32 ± 0.45 and 4.25 ± 0.16, respectively). These differences fully characterize the seriousness of movement limitations at the ankle joint

### Comparison of results using foot plate angle and GM relative length

ANOVA showed significant main effects (factors: footplate angle and experimental group) on externally applied moment, as well as significant interaction between these factors. The biggest difference was found for foot plate angles measured at +4 Nm, being 27° less dorsal flexed in SCP children. In addition, ROM of the foot plate between externally applied moments of 0 Nm to +4 Nm was 6° smaller in SCP children (mean ± SE ∆*φ*_*fp*_ = 17.5 ± 2.2° in SCP children compared to ∆*φ*_*fp*_ = 23.5 ± 1.1° in TD children).

**Figure 6:**
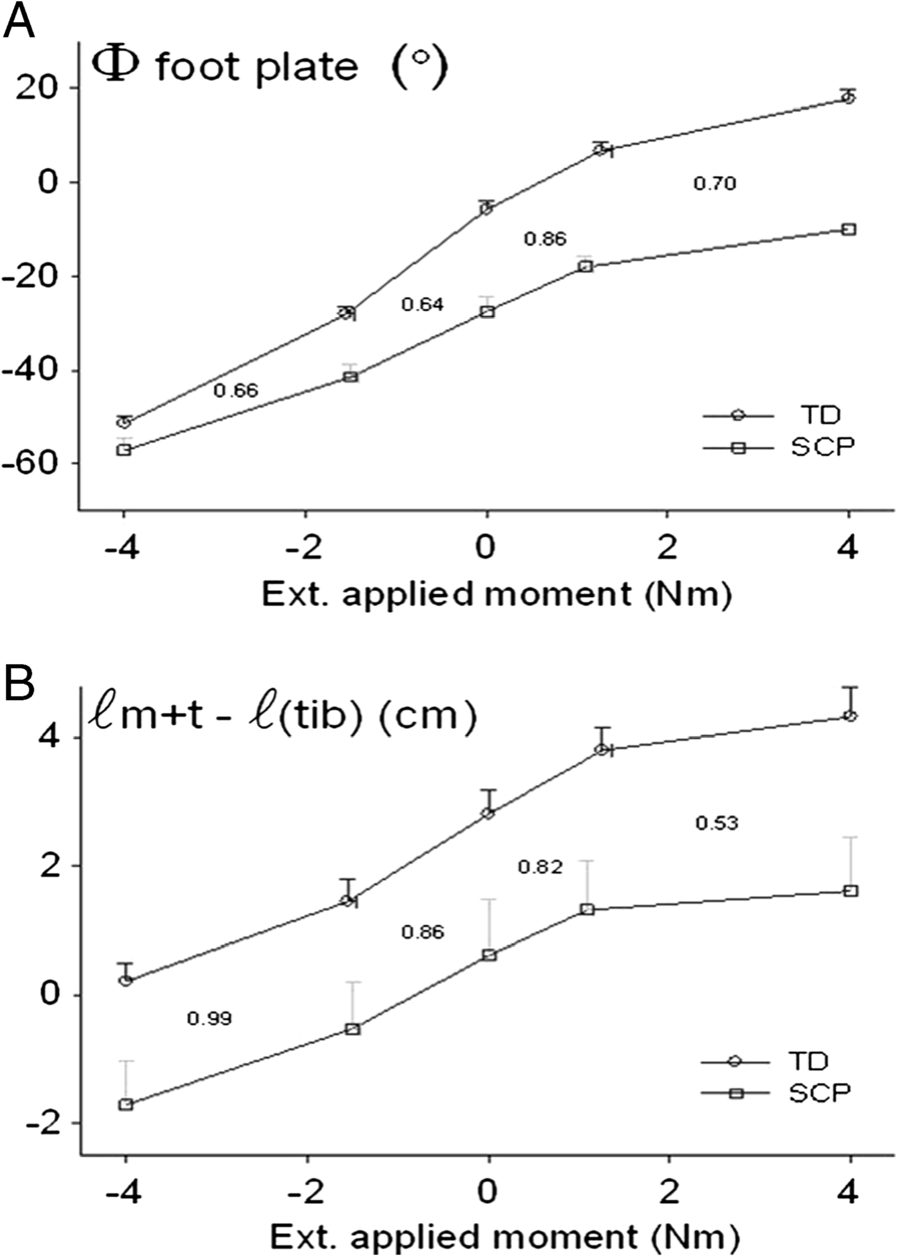
**TD-SCP comparisons for foot plate angle, as well as medial gastrocnemius relative length. A**. Foot plate angle with the horizontally positioned tibia as function of externally exerted moment. Note the converging SCP and TD values towards peak plantar flexion and increasing differences toward dorsal flexion. **B**. GM relative length (*ℓ*_*m+t -*_*ℓ*_*(tib)*_), as estimate of talocrural joint angle, is plotted as function of externally exerted moment. Note that TD-SCP differences are less dependent on exerted moment than in (A.) Mean values + SEM are plotted. In both panels the SCP/TD ratio of slopes of the lines connecting data points are shown.

Figure 6 shows foot plate angle and GM relative length as function of externally applied moment. Note that all curves (in A and B) meet the expectation of steeper slopes (i.e. more movement for a given change of moment) in the middle range compared to both extremes. However at dorsal flexion range (1–4 Nm), the SCP/TD ratio of slopes for the GM relative length curve is much lower (0.53) than that of the foot plate angle (0.70, ratio values also plotted in Figure 6). This is an indication that in the SCP children there is actually more resistance to movement at the talocrural joint, than one would estimate from the foot plate rotation (as is commonly done clinically). This is explained by internal foot deformation as shown above. Note however, that for the peak plantar flexion range (> - 1.6 Nm) the TD and SCP curves for foot plate angle seem to converge, while the curves for GM relative angle (as estimate for talocrural joint angle) are parallel. This suggests that, also for the plantar flexion range, internal foot deformation plays an important role in causing changes in foot plate angle to be an inadequate estimator of angular changes at the talocrural joint.

It is concluded that, for the full movement range studied, internal foot deformation particularly within the mid and forefoot causes rotation within the talocrural joint to be bigger than rotation of the foot plate when moving in plantar flexion direction and to be smaller when moving in dorsal flexion direction.

## Discussion

Comparisons were made for medial gastrocnemius muscle-tendon complex length between TD and SCP children over a range of footplate angles, while the externally applied moment, necessary for bringing the foot plate to the desired position was measured simultaneously. A major finding of this study is that imposed changes of foot plate angle underestimate angular changes in the talocrural joint when moving in plantar flexion direction and overestimate them when moving in dorsal flexion direction. Therefore, we conclude that the use of foot plate angle, usual for studies on human subjects and patients, for SCP children is not adequate to reflect conditions at the ankle joint. That fact became evident by sizeable discrepancies in muscle-tendon complex length between the groups. An effect remains even if effects of differences in skeletal dimensions (c.f. [12,20-22]) on muscle-tendon complex length are dealt with by normalizing for tibia length.

We have provided some evidence that this is caused by movements and deformations within the joints of the foot. Due to the fact that we had access only to X-ray images of one SCP child and none of the TD children prevents us from drawing more general conclusions on a quantitative basis. However, it should be noted that such movements within the foot were reported previously [23], and these authors of the Tardieu-Tabary group also suggested consequences for biomechanics and surgical results for spastic triceps surae.

Note also that recent work from Kawakami‘s group [24] reports substantial deformation of the foot and its effects on m. soleus length for typically developed young adults, comparing passive conditions at different foot plate angles to those during isometric contraction, albeit at considerably higher levels of muscular activity (30-80% MVC) than in our present work. That work constitutes an important contribution to this area of study, but it is surprising that they fail to draw the conclusion, at least explicitly, that foot plate angle is not a valid estimator of talocrural joint angle, and persist in referring the foot plate angle as ankle angle.

If such deformations within the foot are sizable, it will cause invalid comparisons between length- and geometry variables of GM, as well as other muscles, compared at equal foot plate or foot sole angles. We expect that this effect may have contributed to contradicting and confusing results reported in literature for SCP to TD comparison. For example, for SCP children at resting foot sole angles with the tibia (relaxed position of the foot with no external moment applied whatsoever), GM muscle belly, was reported to be a little short relative to typically developing (TD) peers (e.g. [21,22,25]) and that GM tendon to be longer [26]. Based on both animal experimentation and in human subjects, such muscle belly shortness has been presumed to originate from shorter muscle fibers or fascicles (e.g. [5,8]). More recently, using ultrasound imaging, for GM in SCP children, reduced fascicle lengths were reported as well [12,26]. In contrast, compared to TD peers, at so called “resting foot sole angles” (no additional moments exerted), similar fascicle lengths were reported despite the presence of reduced muscle belly length in SCP of similar mean age [21,27] reported similar results at several foot sole angles).

More recent work, combining measurements of moments exerted at the ankle with measurements of calf muscle fascicle and tendon length and pennation angle obtained using 2-D imaging [7-9,11] is very likely to be affected also, by not considering deformation occurring within the foot.

An additional confounding factor may have been a lack of standardization of ultrasound research techniques, since it has been shown that errors of estimation regarding geometrical variables may occur in 2-D imaging [16] due to possible inadequate positioning of the ultrasound probe.

### Potentially altered relative positions of muscle

It should be noted that for SCP subjects, particularly with diplegia having typically planovalgus feet, effects of foot deformation may cause changes in relative positions of m. triceps surae and other muscles with respect to each other and to non-muscular tissues, and cause them to be different from those of the TD subjects. In animal experimentation, changes in muscular relative position have been shown to be an important co-determining variable determining where force generated within a muscle will be actually exerted (for reviews see, [28-30]). Note that experimental evidence regarding these phenomena involving myofascial force transmission were obtained also in human patients undergoing surgery to counteract effects of spastic paresis in arm muscle [31,32]. Recent in vivo studies applying MRI techniques in lower leg muscles of healthy young adults confirmed results compatible with the ideas on effects of muscular relative position [33,34]. On the bases of studies, as cited above, the hypothesis has been proposed [29] that the inability of SCP patients to move their afflicted joints away from a characteristic (dorsal) flexed position is caused by force from antagonistic muscles being transmitted onto agonistic flexor muscles. Further experimental work is essential to deal with these issues.

At the ankle of SCP children, on application of dorsal flexion moments, all plantar flexor muscles crossing the talocrural joint are lengthened less at that joint, but the effect is largest for soleus and gastrocnemius muscles due to their very long moment arm. Therefore, if no compensatory movements would occur (at the joints of mid- and forefoot), the whole foot would be in more plantar flexed position. It is obvious that effects of the foot plate (in our present study), but also similar effects of the floor during more natural loading, do not favor such mid- and forefoot positioning and will cause compensatory dorsal flexion and other more complex movements (i.e. not simple rotations) at the joints of mid- and forefoot. These compensatory movements within the mid- and forefoot affect lengths of all muscles also crossing the ankle joints, except for m. triceps surae, which will only be affected by deformation within the hind-foot (i.e. relative movement of talus and calcanus).

Such altered relative positions of muscles in SCP, potentially may cause changes in patterns of myofascial and myotendinous force transmission. Due to the complexity of movements, particularly in SCP, changes of length and related relative position are hard to predict in detail, so that a systematic study using in vivo imaging is indicated for both SCP and TD subjects. Particularly, for SCP patients this may be difficult, as the need to place such children in a confining bore of an MRI device may limit clinical application, due to anxiety effects on muscle excitation.

### Limitations of this study

In addition to an important limitation mentioned in the introduction regarding the relation between joint moment and GM force, and the minimal number of X-rayed subjects, there are other factors to discuss.

1) We have reported our data on the basis of external moments applied at the interface between torque wrench and foot fixation. Ideally, one would like to know the net moment at the talocrural joint to relate that to muscular variables. However, this net moment can only be estimated globally, with the use of mean data from the literature, concerning foot mass, as well as foot size, in addition to the known apparatus mass. All such factors affect the net ankle moment. Using moment balance equations for the foot, foot-fixation and torque wrench as one free-body (see supplement of [17]), it was calculated that for peak dorsal flexion condition, estimated ankle moment differs most from the external moment actually exerted (i.e. by +1.9 Nm). However, this value did not differ between the groups. Therefore, we preferred to report measured externally applied moment, on the foot plate rather than the estimated net moment at the talocrural joint.

2) As yet for *in vivo* measurements of muscle variables, it is impossible to obtain a valid reference length, such as muscle optimum length or a comparable reference at a specific (mean) sarcomere length, at which muscle variables can be compared truly fairly.

3) Further assessment necessary for new estimates of ankle joint changes using GM relative length. Obviously, GM muscle relative length is not an exact measurement of talocrural joint angles, but only an estimate for them, since it does not take into account such variables as for example moment arm. However, for the present subjects, since it is not affected by foot deformation, it is a better estimate for rotation at the talo-crural joint than foot plate angle. It has an additional potential advantage that it could be used in a clinical setting, even if ultrasound imaging is not available, as it is conceivable that estimation of muscle-tendon length and tibia length at the skin surface could work as well. However, since our present group of SCP subjects has been selected from a much bigger group of patients, using a limited age range as a criterion, it needs to be checked if GM relative length will work as well for groups of SCP patients with a more extended range of ages, where differences in moment arms will be more prevalent.

## Conclusion

Since in SCP children, a sizable part of the rotation of the foot (or foot plate) derives from deformation of the foot rather than from rotation at the talocrural joint it is concluded that foot (plate) angle is not a good estimator of rotation at the talocrural joint.

Therefore, particularly in SCP children with diplegia with planovalgus feet the diagnosed decreased dorsal ROM of the ankle joint is likely to be more serious than judged from range of foot (plate) angles.

## Competing interests

The authors declare that they have no competing interests.

## Authors’ contributions

PAH participated in the conception and design of the study, data analysis and drafting of the manuscripts. MRB performed the experiments and data acquisition, participated in the conception and design of the study, data analysis and drafting of the manuscript. JH participated in the conception and design of the study and revision of the manuscript. RTJ participated in the conception and design of the study, data analysis and drafting of the manuscript. JGB participated in the conception and design of the study, data analysis and revision of the manuscript. All the authors read and approved the final manuscript.

## Pre-publication history

The pre-publication history for this paper can be accessed here:

http://www.biomedcentral.com/1471-2474/14/365/prepub

## References

[B1] Bax M Goldstein M Rosenbaum P Leviton A Paneth N Dan B Proposed definition and classification of cerebral palsy Dev Med Child Neurol 2005 14 571 576 10.1017/S001216220500112X 16108461

[B2] Odding E Roebroeck ME Stam HJ The epidemiology of cerebral palsy: incidence, impairments and risk factors Disabil Rehabil 2006 14 183 191 10.1080/09638280500158422 16467053

[B3] Himmelmann K Hagberg G Uvebrant P The changing panorama of cerebral palsy in Sweden. X. Prevalence and origin in the birth-year period 1999–2002 Acta Paediatr 2010 14 1337 1343 10.1111/j.1651-2227.2010.01819.x 20377538

[B4] Nordmark E Hagglund G Lauge-Pedersen H Wagner P Westbom L Development of lower limb range of motion from early childhood to adolescence in cerebral palsy: a population-based study BMC Med 2009 14 65 10.1186/1741-7015-7-65 19863779 PMC2774339

[B5] Tardieu C Huet De La Tour E Bret MD Tardieu G Muscle hypoextensibility in children with cerebral palsy: clinical and experimental observations Arch Phys Med Rehabil 1982 14 97 102 7073456

[B6] Barrett RS Lichtwark GA Gross muscle morphology and structure in spastic cerebral palsy: a systematic review Dev Med Child Neurol 2010 14 794 804 10.1111/j.1469-8749.2010.03686.x 20477832

[B7] Zhao H Wu Y-N Hwang M Ren Y Gao F Gaebler-Spira D Zhang L-Q Changes of calf muscle-tendon biomechanical properties induced by passive stretching and active movement training in children with cerebral palsy J Appl Physiol 2011 14 435 442 10.1152/japplphysiol.01361.2010 21596920 PMC3154697

[B8] Barber L Barrett R Lichtwark G Passive muscle mechanical properties of the medial gastrocnemius in young adults with spastic cerebral palsy J Biomech 2011 14 2496 2500 10.1016/j.jbiomech.2011.06.008 21762920

[B9] Hoang PD Gorman RB Todd G Gandevia SC Herbert RD A new method for measuring passive length-tension properties of human gastrocnemius muscle in vivo J Biomech 2005 14 1333 1341 10.1016/j.jbiomech.2004.05.046 15863118

[B10] Zhao H Yi-Ning W Jie L Yupeng R Gaebler-Spira DJ Zhang L-Q Changes of calf muscle-tendon properties due to stretching and active movement of children with cerebral palsy – a pilot study 2009 Engineering in Medicine and Biology Society, 2009 Annual International Conference of the IEEE Date of Conference: 3–6 Sept 2009 5287 5290 10.1109/IEMBS.2009.5333518 19964117

[B11] Gao F Zhao H Gaebler-Spira D In vivo evaluations of morphologic changes of gastrocnemius muscle fascicles and Achilles tendon in children with cerebral palsy Am J Phys Med Rehabil 2011 14 364 371 10.1097/PHM.0b013e318214f699 21765255

[B12] Mohagheghi AA Khan T Meadows TH Giannikas K Baltzopoulos V Maganaris CN In vivo gastrocnemius muscle fascicle length in children with and without diplegic cerebral palsy Dev Med Child Neurol 2008 14 44 50 18173630 10.1111/j.1469-8749.2007.02008.x

[B13] Palisano R Rosenbaum P Walter S Russell D Wood E Galuppi B Development and reliability of a system to classify gross motor function in children with cerebral palsy Dev Med Child Neurol 1997 14 214 223 9183258 10.1111/j.1469-8749.1997.tb07414.x

[B14] Bénard MR Jaspers RT Huijing PA Becher JG Harlaar J Reproducibility of hand-held ankle dynamometry to measure altered ankle moment-angle characteristics in children with spastic cerebral palsy Clin Biomech (Bristol, Avon) 2010 14 802 808 10.1016/j.clinbiomech.2010.04.010 20541856

[B15] Prager R Rholing RN Gee AH Berman L Rapid calibration for 3-D freehand ultrasound Ultrasound Med Biol 1998 14 855 869 10.1016/S0301-5629(98)00044-1 9740387

[B16] Bénard MR Becher JG Harlaar J Huijing PA Jaspers RT Anatomical information is needed in ultrasound imaging of muscle to avoid potentially substantial errors in measurement of muscle geometry Muscle Nerve 2009 14 652 665 10.1002/mus.21287 19291798

[B17] Bénard MR Harlaar J Becher JG Huijing PA Jaspers RT Effects of growth on geometry of gastrocnemius muscle in children: a 3-dimensional ultrasound analysis J Anat 2011 14 388 402 10.1111/j.1469-7580.2011.01402.x 21635250 PMC3171775

[B18] Freriks B Hermens H Disselhorst-Klug C Rau G Merletti R Hermens H The recommendations for sensors and sensor placement procedures for surface electromyography SENIAM 8; European Recommendations for Surface Electromyography 1999 Enschede: Roessingh Research and Development B.V 13 54

[B19] Bénard MR Analysis of 3-D Ultrasound of Calf Muscle Geometry in Children: Growth, Spasticity, Mechanisms and Treatment. Doctoral dissertation. ISBN 978-90-865-9582-2 2011 Amsterdam: Vrije Universiteit Amsterdam

[B20] Barber L Hastings-Ison T Baker R Barrett R Lichtwark G Medial gastrocnemius muscle volume and fascicle length in children aged 2 to 5 years with cerebral palsy Dev Med Child Neurol 2011 14 543 548 10.1111/j.1469-8749.2011.03913.x 21506995

[B21] Malaiya R McNee AE Fry NR Eve LC Gough M Shortland AP The morphology of the medial gastrocnemius in typically developing children and children with spastic hemiplegic cerebral palsy J Electromyogr Kinesiol 2007 14 657 663 10.1016/j.jelekin.2007.02.009 17459729

[B22] Oberhofer K Stott NS Mithraratne K Anderson IA Subject-specific modelling of lower limb muscles in children with cerebral palsy Clin Biomech 2010 14 88 94 10.1016/j.clinbiomech.2009.09.007 19836868

[B23] Tardieu C Bret MD Colbeau-Justin P Huet De L Tour E Relationship of triceps surae torques to photographed tibia-calcaneum angles in man (II) Eur J Appl Physiol Occup Physiol 1977 14 153 161 10.1007/BF00421701 902656

[B24] Iwanuma S Akagi R Hashizume S Kanehisa H Yanai T Kawakami Y Triceps surae muscle–tendon unit length changes as a function of ankle joint angles and contraction levels: the effect of foot arch deformation J Biomech 2011 14 2579 2583 10.1016/j.jbiomech.2011.07.003 21831380

[B25] Fry NR Gough M Shortland AP Three-dimensional realisation of muscle morphology and architecture using ultrasound Gait Posture 2004 14 177 182 10.1016/j.gaitpost.2003.08.010 15336288

[B26] Wren TA Cheatwood AP Rethlefsen SA Hara R Perez FJ Kay RM Achilles tendon length and medial gastrocnemius architecture in children with cerebral palsy and equinus gait J Pediatr Orthop 2010 14 479 484 10.1097/BPO.0b013e3181e00c80 20574267

[B27] Shortland AP Harris CA Gough M Robinson RO Architecture of the medial gastrocnemius in children with spastic diplegia Dev Med Child Neurol 2002 14 158 163 10.1017/S0012162201001864 12005316

[B28] Huijing PA Epimuscular myofascial force transmission between antagonistic and synergistic muscles can explain movement limitation in spastic paresis J Electromyogr Kinesiol 2007 14 708 724 10.1016/j.jelekin.2007.02.003 17383897

[B29] Huijing PA Epimuscular myofascial force transmission: a historical review and implications for new research International Society of Biomechanics Muybridge Award Lecture, Taipei, 2007 J Biomech 2009 14 9 21 10.1016/j.jbiomech.2008.09.027 19041975

[B30] Maas H Sandercock TG Force transmission between synergistic skeletal muscles through connective tissue linkages J Biomed Biotechnol 2010 14 575672 20396618 10.1155/2010/575672 PMC2853902

[B31] Kreulen M Smeulders MJ Hage JJ Huijing PA Biomechanical effects of dissecting flexor carpi ulnaris J Bone Joint Surg 2003 14 856 859 12931805

[B32] Smeulders MJ Kreulen M Myofascial force transmission and tendon transfer for patients suffering from spastic paresis: a review and some new observations J Electromyogr Kinesiol 2007 14 644 656 10.1016/j.jelekin.2007.02.002 17369052

[B33] Huijing PA Yaman A Ozturk C Yucesoy CA Effects of knee joint angle on global and local strains within human triceps surae muscle: MRI analysis indicating in vivo myofascial force transmission between synergistic muscles Surg Radiol Anat 2011 14 869 879 10.1007/s00276-011-0863-1 21912991 PMC3224220

[B34] Yaman A Ozturk C Huijing PA Yucesoy CA MRI assessment of mechanical interactions between human lower leg muscles in vivo J Biomech Eng 2013 14 91003 91009 10.1115/1.4024573 23722229

